# Mechanochemical Synthesis of a TiO_2_-Containing Biogenic Hydroxyapatite Ceramic Composite: Balancing Antibiofilm Efficacy and Fibroblast Cytocompatibility

**DOI:** 10.3390/jfb17090426

**Published:** 2026-08-24

**Authors:** Dennys Fernández-Conde, Eneftali Flores-García, Tushar Janardan Pawar, Angélica M. Castillo-Paz, José Rafael Alanis-Gómez, Mario E. Rodríguez-García, Enrique Delgado-Alvarado, Fabiola Hernández-Rosas, Rafael Ramírez-Bon

**Affiliations:** 1 Centro de Investigación y de Estudios Avanzados del IPN, Unidad Querétaro, P.O. Box 1-798, Querétaro 76001, Mexico; dennys.fernandez@upq.edu.mx (D.F.-C.); eneftali.flores@cinvestav.mx (E.F.-G.); angelica.castillo@cinvestav.mx (A.M.C.-P.); 2 Dirección de Ingeniería Mecatrónica, Universidad Politécnica de Querétaro, Carretera Estatal 420 S/N, El Marqués 76240, Querétaro, Mexico; tushar.pawar@anahuac.mx; 3 Centro de Investigación, Universidad Anáhuac Querétaro, Circuito Universidades I, Fracción 2 S/N, Zibatá, El Marqués 76246, Querétaro, Mexico; 4 Escuela de Ingeniería Biomédica, División de Ingeniería, Universidad Anáhuac Querétaro, Circuito Universidades I, Fracción 2 S/N, Zibatá, El Marqués 76246, Querétaro, Mexico; jose.alanis@anahuac.mx; 5 División de Investigación y Posgrado, Facultad de Ingeniería, Universidad Autónoma de Querétaro, Querétaro 76010, Mexico; 6 Departamento de Nanotecnología, Centro de Física Aplicada y Tecnología Avanzada, Universidad Nacional Autónoma de México, Campus Juriquilla, Querétaro 76230, Mexico; mario.rodriguez@unam.mx; 7 Micro and Nanotechnology Research Center, Universidad Veracruzana, Blvd. Av. Ruiz Cortines No. 455 Fracc. Costa Verde, Boca del Río, Veracruz 94294, Mexico; endelgado@uv.mx; 8 Facultad de Química, Universidad Autónoma de Querétaro, Querétaro 76010, Mexico

**Keywords:** biogenic hydroxyapatite, bovine hydroxyapatite, TiO_2_ ceramic composite, rutile, antimicrobial activity, antibiofilm activity, fibroblast cytocompatibility, infection-resistant biomaterials

## Abstract

Implant-associated infections remain a major challenge in bone-related biomedical applications, where bacterial colonization and biofilm formation can compromise tissue integration and clinical performance. This study reports the mechanochemical synthesis, physicochemical characterization, antimicrobial activity, antibiofilm performance, and short-term fibroblast cytocompatibility of a TiO_2_-containing bovine-derived biogenic hydroxyapatite ceramic composite (BHAp-TiO_2_). The composite was prepared by high-energy mechanical milling using 10 wt% TiO_2_ and characterized by X-ray diffraction, Rietveld refinement, Raman spectroscopy, Fourier-transform infrared spectroscopy, scanning electron microscopy, and energy-dispersive X-ray spectroscopy. XRD/Rietveld analysis identified a multiphase ceramic composite composed of hydroxyapatite, whitlockite, and rutile TiO_2_, with no evidence of Ti^4+^ substitution into the hydroxyapatite lattice or detectable anatase within the XRD/Rietveld detection limit. SEM-EDS confirmed the granular agglomerated morphology of the powders and the elemental presence of Ti in BHAp-TiO_2_. Compared with pristine BHAp, BHAp-TiO_2_ produced a concentration-dependent reduction in AlamarBlue^®^-derived bacterial metabolic activity against five clinically relevant planktonic strains. At 200 µg/mL, residual metabolic activity decreased to 10.90–32.90%, depending on the bacterial species, with the strongest response observed for *Escherichia coli*. In crystal violet assays, BHAp-TiO_2_ markedly inhibited *Pseudomonas aeruginosa* biofilm biomass, reaching 91.9 ± 3.4% inhibition at 200 µg/mL. In NIH/3T3 fibroblasts, BHAp-TiO_2_ preserved short-term cytocompatibility after 24 h of direct exposure within the 0.1–100 µg/mL range, with MTT- and AlamarBlue^®^-derived responses remaining close to or above the 80% cytotoxicity limit. Overall, BHAp-TiO_2_ is best interpreted as a rutile TiO_2_-containing biogenic calcium phosphate ceramic composite with enhanced antimicrobial and antibiofilm performance while maintaining short-term fibroblast cytocompatibility under the evaluated conditions.

## 1. Introduction

Implant-associated infections (IAIs) represent one of the most persistent and costly complications in modern orthopedics, dentistry, and related biomedical fields. Despite significant advancements in surgical techniques and sterilized environments, these infections are frequently initiated by the rapid adhesion of opportunistic pathogens to abiotic surfaces [1,2]. Once attached, bacteria undergo a phenotypic shift, developing into complex, self-produced extracellular polymeric substance (EPS) matrices known as biofilms. These biofilms serve as a formidable shield, enabling immune evasion and markedly increasing bacterial tolerance to systemic antibiotics and local disinfectants compared to their planktonic counterparts. The clinical impact is further amplified by the dominance of high-risk pathogens with strong biofilm-forming capacities, particularly Gram-positive species such as *Staphylococcus aureus* and *Staphylococcus epidermidis*, as well as Gram-negative species like *Pseudomonas aeruginosa*, which are notoriously difficult to eradicate once a mature biofilm is established on a medical device [1,3,4].

Within this context, there is a growing clinical consensus that infection control cannot rely solely on systemic antibiotic prophylaxis, which often fails to reach effective concentrations at the implant interface and contributes to the global crisis of antimicrobial resistance. Rather, the biomaterials themselves should be engineered as the first line of defense, designed to intrinsically resist bacterial colonization while simultaneously maintaining or enhancing compatibility with host tissues [1,2]. This dual requirement, antimicrobial efficacy paired with high bioactivity, has driven the search for advanced functional ceramics that can integrate into the physiological environment of bone while reducing bacterial adhesion and biofilm development [5,6].

Hydroxyapatite (HAp, Ca_10_(PO_4_)_6_(OH)_2_) has long been recognized as a highly relevant calcium phosphate ceramic in biomedical applications because its chemical composition and crystalline structure closely resemble the mineral phase of human bone. Its inherent osteoconductivity and ability to support osseointegration make it an ideal candidate for bone grafts, scaffolds, and bioactive coatings on metallic implants [7,8,9].

However, conventional synthetic HAp is not intrinsically antimicrobial. When exposed to contaminated surgical environments or hematogenous seeding, HAp surfaces may permit bacterial adhesion and subsequent biofilm formation [7,8]. Consequently, a significant design challenge in biomaterials science is to impart antibacterial functionality to HAp-based structures without undermining their osteoconductive and cytocompatible behavior.

Ion substitution, surface functionalization, and composite formation have emerged as versatile strategies to tune physicochemical and biological performance of apatite-based materials. Owing to the chemical flexibility of the apatite lattice, functional ions can modify material solubility, surface charge, microstructure, and biological interactions [10]. In addition to conventional antimicrobial elements such as silver, copper, zinc, magnesium, and strontium, rare-earth elements have been investigated in hydroxyapatite-containing systems because of their potential to modify interfacial chemistry and biological performance [9]. In this context, cerium- and samarium-containing compounds provide relevant examples of the interaction between rare-earth elements and mineralized calcium phosphate substrates. Kopp et al. demonstrated that cerium(III) and samarium(III) nitrates precipitated on human enamel independently of the presence of a salivary pellicle and modified its surface elemental composition, including the Ca/P ratio [10]. These findings indicate that the biological performance of rare-earth-modified calcium phosphate systems depends on the specific element, its chemical form, concentration, and interaction with the mineral substrate; therefore, their potential antimicrobial functionality should be considered together with cytocompatibility and material-specific interfacial effects [9,10,11]. This has motivated continued interest in alternative ceramic phases capable of balancing antimicrobial activity, structural stability, and acceptable cytocompatibility [11].

Titanium dioxide (TiO_2_) is a particularly attractive candidate for this role. Known for its excellent chemical stability and low solubility, TiO_2_ has been studied extensively for its antimicrobial activity and biomedical surface applications [12,13]. Traditionally associated with photocatalytic mechanisms, TiO_2_ may also exert antibacterial effects through surface-mediated pathways, including membrane perturbation, altered bacterial adhesion, and oxidative stress generation [12]. Furthermore, TiO_2_-containing calcium phosphate systems have been investigated as multifunctional biomaterials that combine the bioactivity of apatite with the antimicrobial or antibiofilm contribution of titanium oxide phases [14,15]. However, the biological response of HAp-TiO_2_ systems depends strongly on phase composition, crystallinity, surface morphology, particle dispersion, and the structural relationship between TiO_2_ and the apatite matrix. Thus, developing TiO_2_-containing hydroxyapatite composites requires rigorous physicochemical characterization to distinguish lattice-level modification from the formation of TiO_2_-containing composite structures [16].

An additional translational advantage can be found in the use of natural-source hydroxyapatite derived from bovine bone (BHAp). Unlike purely synthetic counterparts, BHAp often retains compositional features, carbonate substitution, trace elements, and microstructural characteristics closer to biological apatite, making it a sustainable and scalable starting material for biomedical-grade ceramics [7,16,17]. Despite the increasing interest in TiO_2_-containing calcium phosphate systems, there remains a critical need for comprehensive studies that link specific physicochemical properties, such as phase composition, lattice parameters, elemental distribution, and surface morphology, with biological outcomes across clinically relevant pathogens. Specifically, there is a lack of detailed data regarding how TiO_2_-containing BHAp composites influence the transition from planktonic bacterial inhibition to the more challenging prevention of biofilm formation. Because early reductions in planktonic growth do not always accurately predict performance against surface-attached bacterial communities, characterizing antibiofilm activity is essential for the development of infection-resistant biomaterials [2,18]. Moreover, evaluating these materials against both Gram-positive and Gram-negative species, such as *Staphylococcus aureus*, *Staphylococcus epidermidis*, *Enterococcus faecalis*, *Escherichia coli*, and *Pseudomonas aeruginosa*, is necessary to provide meaningful insight into the strain-dependent biological response required for biomedical applications [1,3].

In the present study, we address these challenges by reporting the synthesis and comprehensive evaluation of a TiO_2_-containing bovine-derived hydroxyapatite composite (BHAp-TiO_2_ composite) formulated with 10 wt% TiO_2_ obtained through high-energy mechanochemical milling. This processing route was selected to promote intimate contact and dispersion of TiO_2_ within the biogenic apatite-based powder. The resulting material was subjected to systematic physicochemical characterization using X-ray diffraction (XRD), Rietveld refinement, Raman spectroscopy, Fourier-transform infrared spectroscopy (FTIR), scanning electron microscopy (SEM), and energy-dispersive X-ray spectroscopy (EDS) to assess phase composition, crystallographic parameters, vibrational features, morphology, and elemental distribution. To evaluate biological functionality, antimicrobial performance was assessed against a panel of five bacterial strains, and antibiofilm activity was evaluated using *Pseudomonas aeruginosa* as a model biofilm-forming pathogen. Finally, short-term in vitro cytocompatibility was evaluated in NIH/3T3 fibroblasts to determine whether the antimicrobial functionality was accompanied by an acceptable mammalian cell response.

Accordingly, this study tested two principal null hypotheses: (H0_1_) the distributions of bacterial metabolic activity and *Pseudomonas aeruginosa* biofilm biomass do not differ between pristine BHAp and BHAp–TiO_2_ across the evaluated concentrations; (H0_2_) the distributions of NIH/3T3 fibroblast viability and metabolic activity do not differ between pristine BHAp and BHAp–TiO_2_ after 24 h of direct exposure across the evaluated concentrations.

## 2. Materials and Methods

### 2.1. Synthesis of TiO_2_-Containing Biogenic Hydroxyapatite Composite

The bovine-derived hydroxyapatite (BHAp) used in this study was obtained via a biomimetic procedure adapted from previously reported protocols for the preparation and physicochemical characterization of bovine bone-derived hydroxyapatite scaffolds [19,20].

Femoral bovine bones were obtained postmortem as by-products from a registered local slaughterhouse in Querétaro, Mexico. No animals were sacrificed specifically for this study, and no experimental procedures were performed on live animals. Therefore, the study did not involve animal experimentation; the bovine bone material was used exclusively as an abattoir-derived biological by-product.

Briefly, the bovine bone epiphyses were sectioned into approximately 2 cm slices and subjected to hydrothermal treatment at 180 °C for 3 h to remove residual organic components. The treated bone fragments were dried at 95 °C for 48 h to increase brittleness, manually ground using a metallic mortar, and subsequently sieved. The resulting powder was calcined at 700 °C for 24 h to obtain biogenic hydroxyapatite powder.

The TiO_2_-containing BHAp composite was prepared through a mechanochemical grinding–dispersion process. Based on the experimental design, 10 wt% TiO_2_ powder (anatase, Sigma-Aldrich, 99%) was mixed with 90 wt% biogenic BHAp powder. The powders were initially homogenized manually in an agate mortar and then subjected to high-energy mechanical milling for 3 h using a SPEX SamplePrep 8000M Mixer/Mill (Azzota Scientific, Claymont, DE, USA). This process was performed to promote particle-size homogenization and close interfacial contact between TiO_2_ and the biogenic calcium phosphate-based powder. The resulting composite powder containing 10 wt% TiO_2_ was designated as BHAp-TiO_2_ composite throughout the manuscript. Because the BHAp-TiO_2_ composite was prepared by dry high-energy mechanochemical milling, no pH-controlled liquid medium was involved during the composite formation step. Therefore, pH was not considered a processing variable during the mechanochemical association of BHAp with TiO_2_.

This terminology was selected because the material was designed as a TiO_2_-containing biogenic hydroxyapatite composite, rather than as a substitutionally doped hydroxyapatite. After milling, the powders were allowed to cool naturally to room temperature and stored in desiccators until further physicochemical and biological characterization.

### 2.2. X-Ray Diffraction (XRD) Analysis and Rietveld Refinement

The crystalline phase composition and structural integrity of the synthesized BHAp and BHAp-TiO_2_ composite powders were determined using X-ray diffraction (XRD). Diffraction patterns were recorded on a Rigaku Dmax-2100 diffractometer (Rigaku Corporation, Tokyo, Japan). The system utilized a Cu target radiation source (Kα doublet: 1.5406 Å/1.5444 Å) operating at 30 kV and 20 mA. Measurements were conducted using an optical geometry with parallel beams, maintaining a fixed incident angle of 1° relative to the flat surface of the sample. Data collection was performed over a 2θ range from 5° to 70° with a step size of 0.02°. Phase identification was carried out by comparing the experimental diffractograms with standard reference patterns for hydroxyapatite (PDF#00-009-0432), whitlockite (PDF#00-009-0169), anatase TiO_2_ (PDF#00-021-1272), and rutile TiO_2_ (PDF#00-021-1276).

Rietveld refinement was performed to further evaluate phase composition, lattice parameters, and crystallographic changes associated with the mechanochemical processing of BHAp with TiO_2_. The refinement was used to determine whether the diffraction features were consistent with preservation of the apatite structure, formation of secondary calcium phosphate phases, and/or the presence of titanium oxide crystalline phases. The analysis was not used as sole evidence of substitutional incorporation of Ti^4+^ into the hydroxyapatite lattice.

### 2.3. Raman Spectroscopy

Raman spectroscopy was employed to corroborate the chemical composition and vibrational features of the synthesized powders. Spectra were recorded using a micro-Raman spectrometer Jobin Yvon Dilor Labram II (Longjumeau, Dilor, France) equipped with a He–Ne laser operating at 632.8 nm within the 100–1100 cm^−1^ range. The laser power was maintained at 50 mW for all measurements, which were performed at room temperature using a 50× objective lens Olympus LM Plan Fl (Olympus, Tokyo, Japan). This optical configuration provided localized analysis with high spatial resolution. Acquisition parameters were kept constant across all samples to ensure spectral comparability. Finally, Fityk^®^ software Version 1.3.0 was used to perform deconvolution of the identified vibrational modes in both BHAp and BHAp-TiO_2_ composite powders. The Raman analysis was interpreted considering the characteristic vibrational modes of hydroxyapatite and titanium dioxide polymorphs, including anatase and rutile, to avoid assigning TiO_2_-related bands to a single crystalline phase without complementary structural evidence.

### 2.4. Fourier-Transform Infrared Spectroscopy (FTIR)

Fourier-transform infrared spectroscopy (FTIR) was used to identify functional groups and verify the preservation of characteristic hydroxyapatite vibrations following the formation of the BHAp-TiO_2_ composite. Spectra were acquired at normal incidence using a Perkin-Elmer Frontier spectrometer in attenuated total reflectance (ATR) mode. The analysis was conducted in the range of 4000–600 cm^−1^ with a spectral resolution of 4 cm^−1^. The resulting spectra were evaluated to confirm the presence of PO_4_^3−^ and OH^−^ groups and to detect vibrational shifts or new absorption bands associated with Ti–O and Ti–O–Ti vibrations. The FTIR analysis was interpreted as complementary evidence of possible interfacial interaction or association between TiO_2_ and BHAp, rather than as definitive proof of Ti^4+^ substitution within the hydroxyapatite lattice.

### 2.5. Morphological and Elemental Characterization (SEM and EDS)

The surface morphology and microstructural features of the BHAp and BHAp-TiO_2_ composite powders were examined using scanning electron microscopy JEOL JSM-6010 PLUS/LA (Jeol Ltd., Tokyo, Japan). The system was operated at an accelerating voltage of 20 kV in low-vacuum mode, using secondary electron detection and a working distance (WD) of 10 mm. Low-vacuum conditions were selected to reduce charging artifacts during the analysis of ceramic powders with heterogeneous particle sizes and limited electrical conductivity. To ensure a homogeneous distribution for imaging, the powders were dispersed in ethanol and subjected to 4 min of ultrasonic treatment to minimize particle agglomeration. The suspension was then deposited onto aluminum stubs and secured with conductive carbon adhesive. Before analysis, samples were coated with a thin gold–palladium layer to enhance electrical conductivity and suppress charging effects.

Elemental composition and titanium distribution within the analyzed powder areas were determined using energy-dispersive X-ray spectroscopy (EDS) coupled to the SEM system. Carbon was not excluded from the EDS spectra because carbon-containing signals may originate from carbonate substitutions or adsorbed carbonaceous species associated with biogenic hydroxyapatite, as well as from the conductive carbon adhesive used for sample mounting. Therefore, carbon values were interpreted qualitatively and with caution, rather than as definitive evidence of the intrinsic chemical composition of the composite.

### 2.6. In Vitro Cytocompatibility Assessment by AlamarBlue^®^ and MTT Assays

The in vitro cytocompatibility of BHAp and BHAp-TiO_2_ was evaluated using complementary AlamarBlue^®^ and MTT assays to assess cellular metabolic activity and viability, respectively. NIH/3T3 fibroblast cells ATCC CRL-1658 (American Type Culture Collection, Manassas, VA, USA) were used as a standardized fibroblastic cell model for preliminary cytocompatibility screening. Cells were cultured in Dulbecco’s Modified Eagle Medium (DMEM; Gibco™, Thermo Fisher Scientific, Waltham, MA, USA) supplemented with 10% heat-inactivated fetal bovine serum (FBS; Gibco™, Thermo Fisher Scientific, USA). Cultures were maintained at 37 °C in a humidified atmosphere containing 5% CO_2_ using a Heratherm™ CO_2_ incubator (Thermo Fisher Scientific, USA).

For the experiments, NIH/3T3 fibroblasts were seeded in 24-well plates at an initial density of 1 × 10^4^ cells/well in complete DMEM and incubated until reaching approximately 80% confluence before treatment. Prior to biological testing, BHAp and BHAp-TiO_2_ powders were pretreated using 70% ethanol, allowed to dry under aseptic conditions, and subsequently exposed to ultraviolet (UV) irradiation for 2 h. This combined ethanol/UV pretreatment was performed to reduce potential microbial contamination before direct contact with cell cultures while minimizing possible physicochemical alterations of the powders.

The pretreated powders were dispersed directly in complete DMEM to obtain final concentrations of 0.1, 1, 10, and 100 µg/mL and were applied to cell cultures by direct contact for 24 h. The 24-h exposure period was selected as an initial short-term cytocompatibility screening endpoint to identify acute metabolic or viability alterations induced by direct exposure to the particulate materials. Untreated cells cultured under the same conditions were used as the negative control and represented 100% cell viability or metabolic activity. Blank wells containing culture medium and the corresponding reagent without cells were included for background correction.

Following exposure, cell morphology and adherence were qualitatively monitored using an inverted optical microscope (Olympus CKX53, Olympus Corporation, Tokyo, Japan). Each experimental condition was analyzed in technical triplicate within each assay, and the complete experiment was independently repeated three times on different days. Data are presented as mean ± standard deviation from three independent experiments.

#### 2.6.1. AlamarBlue^®^ Assay

After 24 h of direct exposure to BHAp or BHAp-TiO_2_, the culture medium was replaced with fresh DMEM containing AlamarBlue^®^ reagent at 10% *v*/*v*, and the plates were incubated for 4 h at 37 °C in a humidified atmosphere containing 5% CO_2_. Metabolic activity was quantified by measuring absorbance at 570 and 600 nm using a Multiskan™ FC microplate reader (Thermo Fisher Scientific, USA). The absorbance at 570 nm was used as the primary signal associated with resazurin reduction, whereas absorbance at 600 nm was used as a reference wavelength for dual-wavelength correction.

Relative metabolic activity was calculated according to the following equation:

$$Metabolic\;activity\left(\%\right)=\frac{\left[\left(A_{570}\;treated-A_{600}\;treated\right)-\left(A_{570}\;blank-A_{600}\;blank\right)\right]}{\left[\left(A_{570}\;control-A_{600}\;control\right)-\left(A_{570}\;blank-A_{600}\;blank\right)\right]}\times 100$$

where A_570_ treated and A_600_ treated correspond to the absorbance values of cells exposed to BHAp or BHAp-TiO_2_; A_570_ control and A_600_ control correspond to untreated control cells; and A_570_ blank and A_600_ blank correspond to wells containing culture medium and AlamarBlue^®^ reagent without cells. Results were expressed as percentage of metabolic activity relative to untreated control cells, which were defined as 100% metabolic activity.

#### 2.6.2. MTT Assay

For the MTT assay, after 24 h of direct exposure to BHAp or BHAp-TiO_2_, cells were incubated with MTT solution (5 mg/mL in PBS; Gibco™, Thermo Fisher Scientific, USA) for 2 h at 37 °C to allow formazan crystal formation. After incubation, the supernatant was carefully removed, and the formazan crystals were solubilized in isopropanol under gentle agitation for 1 h in the dark. Absorbance was measured at 570 nm using a Multiskan™ FC microplate reader (Thermo Fisher Scientific, USA).

Cell viability was calculated using the following equation:

$$\mathrm{Cell}\;\mathrm{viability}(\%)=\frac{\left(A_{570}\;treated-A_{570}\;blank\right)}{\left(A_{570}\;control-A_{570}\;blank\right)}\times 100$$

where A_570_ treated corresponds to the absorbance of cells exposed to BHAp or BHAp-TiO_2_; A_570_ control corresponds to untreated control cells; and A_570_ blank corresponds to wells containing culture medium and MTT reagent without cells. Data were expressed as percentage of cell viability relative to untreated control cells, which were defined as 100% viability.

### 2.7. Bacterial Strains and Culture Conditions

The antimicrobial performance of BHAp and BHAp-TiO_2_ was evaluated against a representative panel of clinically relevant Gram-positive and Gram-negative bacterial strains associated with biomaterial-related infections, wound contamination, nosocomial infections, and biofilm formation. The Gram-positive strains included *Staphylococcus aureus* ATCC^®^ 25923, *Staphylococcus epidermidis* ATCC^®^ 14990, and *Enterococcus faecalis* ATCC^®^ 29212. The Gram-negative strains included *Escherichia coli* ATCC^®^ 25922 and *Pseudomonas aeruginosa* ATCC^®^ 27853.

The bacterial panel was selected to include microorganisms with different cell envelope structures, virulence mechanisms, and clinical relevance in biomedical-device-associated infections. *S. aureus* was included due to its relevance in wound infections, osteomyelitis, and implant-associated infections; *S. epidermidis* was selected because of its ability to colonize biomaterial surfaces and form biofilms on medical devices; *E. faecalis* was included as a clinically relevant Gram-positive opportunistic pathogen with high environmental tolerance; *E. coli* was selected as a representative Gram-negative bacillus commonly used in antimicrobial susceptibility assays; and *P. aeruginosa* was included because of its intrinsic antimicrobial tolerance, robust biofilm-forming capacity, and relevance in chronic wound and hospital-acquired infections.

Bacterial strains were cultured aerobically in Mueller–Hinton broth (MHB; BD Difco™, Becton, Dickinson and Company, Sparks, MD, USA). Overnight cultures were incubated at 37 °C using a Heratherm™ microbiological incubator (Model IMH-60, Thermo Fisher Scientific, Waltham, MA, USA). For antimicrobial assays, bacterial cultures were used during the logarithmic growth phase. The bacterial inoculum was adjusted to a turbidity equivalent to a 0.5 McFarland standard, corresponding approximately to 1 × 10^8^ CFU/mL, and subsequently diluted in fresh MHB to obtain a final working inoculum of 5 × 10^5^ CFU/mL, according to Clinical and Laboratory Standards Institute (CLSI) broth microdilution guidelines for aerobic bacteria.

### 2.8. Antimicrobial Activity Assessment by AlamarBlue^®^ Assay

The antimicrobial activity of BHAp and BHAp-TiO_2_ against planktonic bacteria was evaluated using a resazurin-based metabolic activity assay with AlamarBlue^®^ reagent. Prior to biological testing, BHAp and BHAp-TiO_2_ powders were prepared following the same procedure used for the cytocompatibility assays. Briefly, the powders were washed with 70% ethanol, allowed to dry under aseptic conditions, and subsequently exposed to ultraviolet (UV) irradiation for 2 h before being resuspended in Mueller–Hinton broth. Bacterial suspensions were prepared in Mueller–Hinton broth (MHB; BD Difco™, Becton, Dickinson and Company, Sparks, MD, USA) and adjusted to a final concentration of 5 × 10^5^ CFU/mL. Standardized bacterial inocula were dispensed into sterile 96-well cell culture microplates (Corning^®^, Corning, NY, USA). BHAp and BHAp-TiO_2_ powders were independently added to the corresponding wells to obtain final concentrations of 0.1, 1, 10, 100, and 200 µg/mL. Untreated bacterial suspensions were included as bacterial growth controls and represented 100% metabolic activity. Sterile MHB without bacteria was used as the medium blank and sterility control. Wells containing BHAp or BHAp-TiO_2_ suspended in MHB without bacteria were included as material background controls to correct for possible optical interference associated with the particulate materials. Vancomycin (Sigma-Aldrich, Cat. No. 94747, St. Louis, MO, USA) was used as the positive antimicrobial control for Gram-positive strains, specifically *Staphylococcus aureus*, *Staphylococcus epidermidis*, and *Enterococcus faecalis*. Amikacin hydrate (Sigma-Aldrich, Cat. No. A3650, St. Louis, MO, USA) was used as the positive antimicrobial control for Gram-negative strains, specifically *Escherichia coli* and *Pseudomonas aeruginosa.* The microplates were incubated under aerobic conditions for 24 h at 37 °C using a Heratherm™ microbiological incubator (Model IMH-60, Thermo Fisher Scientific, Waltham, MA, USA). After incubation, AlamarBlue^®^ reagent (Invitrogen™, Thermo Fisher Scientific, Waltham, MA, USA) was added to each well at 10% *v*/*v*, according to the manufacturer’s instructions. The plates were further incubated under the same conditions to allow the reduction of resazurin to resorufin by metabolically active bacterial cells. Bacterial metabolic activity was quantified by measuring absorbance at 570 and 600 nm using a Multiskan™ FC microplate reader (Thermo Fisher Scientific, Waltham, MA, USA). The absorbance at 570 nm was used as the primary signal associated with resazurin reduction, whereas absorbance at 600 nm was used as the reference wavelength for dual-wavelength correction. For each condition, medium blanks and material-only background controls were subtracted before calculating bacterial viability.

Bacterial viability was calculated relative to the untreated bacterial growth control using the following equation:

$$Bacterial\;viability\;(\%)=\frac{\left[(A_{570}\;sample\;-\;A_{600}\;sample)\;-\;(A_{570}\;material\;background\;-\;A_{600}\;material\;background)\right]}{\left[(A_{570}\;growth\;control\;-\;A_{600}\;growth\;control)\;-\;(A_{570}\;medium\;blank\;-\;A_{600}\;medium\;blank)\right]}\;\times 100$$

Antimicrobial inhibition was calculated as follows:

Antimicrobial inhibition (%) = 100 − bacterial viability (%)

where A_570_ sample and A_600_ sample correspond to the absorbance values of bacteria exposed to BHAp or BHAp-TiO_2_; A_570_ material background and A_600_ material background correspond to BHAp or BHAp-TiO_2_ suspended in MHB without bacteria; A_570_ growth control and A_600_ growth control correspond to untreated bacterial suspensions; and A_570_ medium blank and A_600_ medium blank correspond to sterile MHB without bacteria. The antimicrobial response was reported as bacterial viability (%) and antimicrobial inhibition (%), as appropriate for each dataset. Because the AlamarBlue^®^ assay is based on metabolic reduction of resazurin and does not directly quantify viable colony counts, log_10_ CFU/mL reductions were not derived from absorbance data. Each experimental condition was analyzed in technical triplicate within each assay, and the complete experiment was independently repeated three times on different days. Data are presented as mean ± standard deviation from three independent experiments.

### 2.9. Biofilm Formation and Inhibition Assay

Biofilm formation and inhibition were evaluated using *Pseudomonas aeruginosa* following a static microtiter-plate assay based on crystal violet staining. *P. aeruginosa* was selected as a targeted antibiofilm model because of its strong biofilm-forming capacity, clinical relevance in chronic and device-associated infections, and frequent use as a reference organism for evaluating antibiofilm activity on abiotic surfaces. Prior to the antibiofilm assay, BHAp and BHAp-TiO_2_ powders were prepared following the same ethanol/UV pretreatment described for the cytocompatibility and antimicrobial assays. Briefly, the powders were washed with 70% ethanol, allowed to dry under aseptic conditions, and subsequently exposed to ultraviolet irradiation for 2 h before being resuspended in Mueller–Hinton broth. Bacterial suspensions were prepared in Mueller–Hinton broth (MHB; BD Difco™, Becton, Dickinson and Company, Sparks, MD, USA) and adjusted to a final concentration of 5 × 10^5^ CFU/mL, as described in Section 2.7. Aliquots of the standardized bacterial suspension were dispensed into sterile 96-well microplates (Corning^®^, Corning, NY, USA). BHAp and BHAp-TiO_2_ powders were independently added to the corresponding wells to obtain final concentrations of 0.1, 1, 10, 100, and 200 µg/mL. Untreated bacterial suspensions were used as biofilm growth controls and represented 100% biofilm formation. Wells containing MHB without bacteria were used as blanks for background correction. The plates were incubated under static aerobic conditions at 37 °C for 24 h using a Heratherm™ microbiological incubator to allow biofilm development. After incubation, the planktonic phase was carefully removed, and the wells were gently washed three times with sterile phosphate-buffered saline (PBS) to remove non-adherent cells. The remaining surface-adhered biofilms were fixed with methanol for 15 min and stained with 1% (*w*/*v*) crystal violet solution for 15 min at room temperature. Excess crystal violet was removed by rinsing the wells with distilled water, and the plates were air-dried. The bound crystal violet was then solubilized using 30% (*v*/*v*) acetic acid. Biofilm biomass was quantified by measuring absorbance at 570 nm using a Multiskan™ FC microplate reader (Thermo Fisher Scientific, Waltham, MA, USA). Blank-corrected biofilm formation was calculated relative to the untreated biofilm growth control using the following equation:

$$Biofilm\;formation\left(\%\right)=\frac{\left(A_{570}\;treated-A_{570}\;blank\right)}{\left(A_{570}\;growth\;control-A_{570}\;blank\right)}\times 100$$

Biofilm inhibition was calculated as follows:

$$Biofilm\;inhibition\left(\%\right)=\frac{\left(A_{570}\;growth\;control\;-\;A_{570}\;blank)\;-\;(A_{570}\;treated\;-\;A_{570}\;blank\right)}{\left(A_{570}\;growth\;control-A_{570}\;blank\right)}\times 100$$

where A_570_ treated corresponds to the absorbance of biofilms exposed to BHAp or BHAp-TiO_2_, A_570_ growth control corresponds to untreated *P. aeruginosa* biofilms, and A_570_ blank corresponds to wells containing MHB without bacteria processed under the same staining and destaining conditions. Untreated biofilm growth controls were defined as 100% biofilm formation and 0% biofilm inhibition. The crystal violet assay was used to quantify total attached biofilm biomass and does not directly distinguish between viable and non-viable bacterial cells. For qualitative assessment, representative stained wells were observed before dye solubilization at 20× magnification using an optical microscope to visualize differences in surface coverage and biofilm architecture. Data are presented as mean ± standard deviation from three independent experiments.

### 2.10. Statistical Analysis

Statistical analyses were performed using IBM SPSS Statistics, version 29.0 (IBM Corp., Armonk, NY, USA). Biological data were obtained from three independent experiments conducted on different days. Each experimental condition was assessed in technical triplicate, and the technical replicates were averaged to obtain one value for each independent experiment; therefore, the independent experiment was considered the statistical unit (*n* = 3 per condition).

No formal a priori sample-size or statistical-power calculation was performed because the biological component of the study was designed as an exploratory in vitro biomaterials-screening investigation. Given the limited number of independent experiments, the underlying data distribution could not be assessed reliably, and normality was therefore not assumed. Data are presented descriptively as mean ± standard deviation to maintain a consistent reporting format across biological assays and figures; this descriptive convention should not be interpreted as evidence of normally distributed data and was not used to determine the selection of the inferential statistical procedures.

Inferential comparisons among multiple groups were performed using the Kruskal–Wallis non-parametric test, followed by Dunn–Bonferroni rank-based post hoc multiple comparisons, as appropriate. All inferential conclusions were based exclusively on these non-parametric procedures. Statistical significance was established at *p* < 0.05.

## 3. Results

### 3.1. Microstructural Characteristics

#### 3.1.1. X-Ray Diffraction (XRD) and Rietveld Refinement

**Figure 1:**
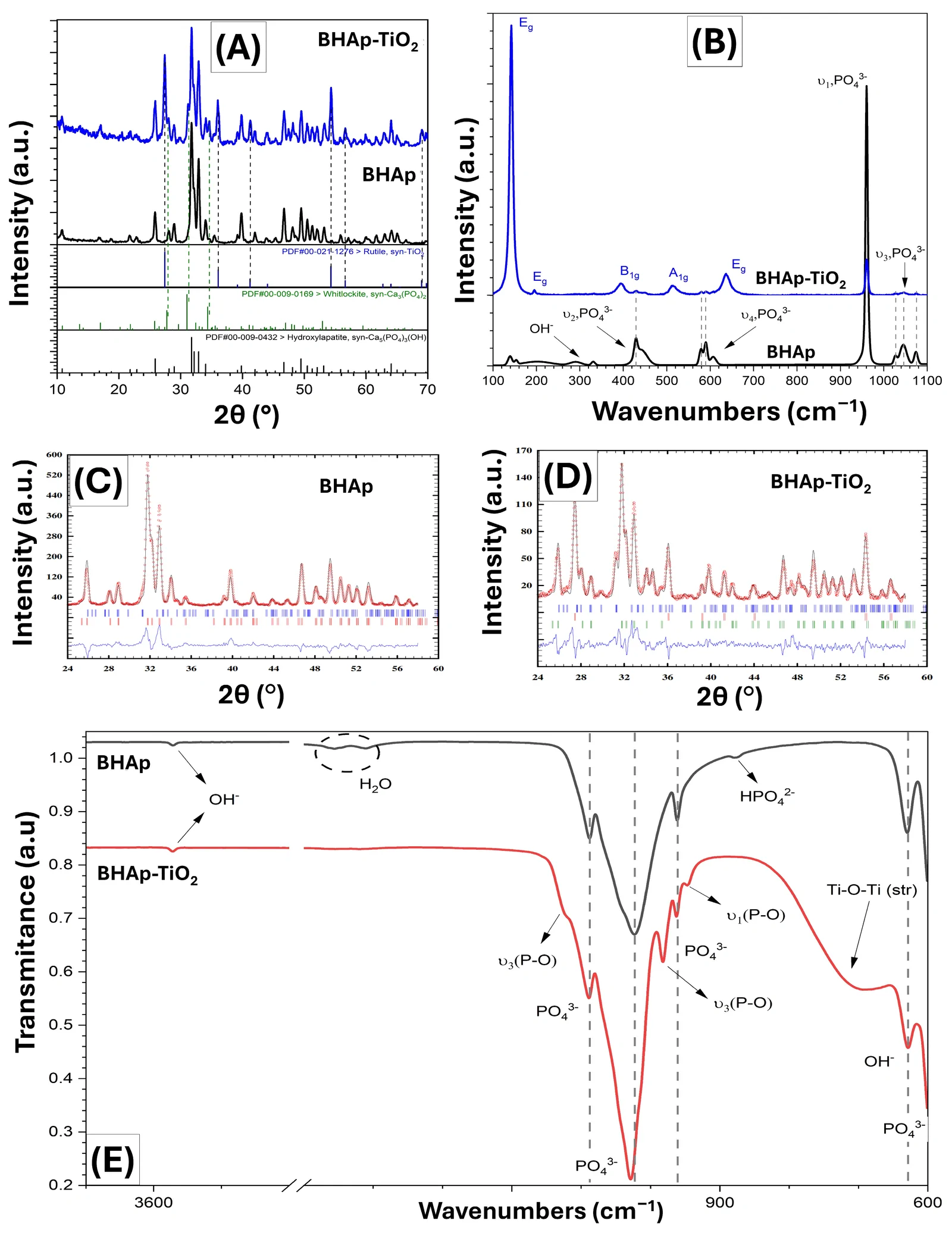
Structural and spectroscopic characterization of BHAp and BHAp-TiO_2_ powders. (**A**) X-ray diffraction patterns of BHAp and BHAp-TiO_2_, shown as black and blue profiles, respectively, including reference reflections for hydroxyapatite, whitlockite, and rutile TiO_2_. The colored reference bars indicate the characteristic Bragg reflection positions of the corresponding crystalline phases, while the gray vertical dashed lines highlight selected characteristic diffraction positions for comparison with the experimental patterns. (**B**) Raman spectra of BHAp (black) and BHAp-TiO_2_ (blue), showing phosphate-related vibrational modes in BHAp and TiO_2_-related vibrational features in BHAp-TiO_2_. Gray vertical dashed lines indicate selected characteristic Raman-band positions used for vibrational assignment. (**C**,**D**) Rietveld refinement profiles of BHAp and BHAp-TiO_2_, respectively, showing the observed and calculated diffraction profiles, the difference curve, and the colored tick marks corresponding to the Bragg reflection positions of the refined crystalline phases. The light-blue curve at the bottom represents the difference between the observed and calculated diffraction profiles. (**E**) FTIR spectra of BHAp (black) and BHAp-TiO_2_ (red), showing phosphate, hydroxyl, carbonate-related, and Ti–O/Ti–O–Ti vibrational contributions. Gray vertical dashed lines indicate selected characteristic absorption-band positions used for spectral assignment. XRD patterns and Raman/FTIR spectra were vertically offset for clarity. The corresponding Rietveld refinement parameters and phase fractions are provided in Supplementary Table S1.

The XRD patterns of BHAp and BHAp-TiO_2_ are shown in Figure 1A, together with the reference patterns used for crystalline phase identification. The BHAp diffractogram showed the characteristic reflections of hydroxyapatite (PDF#00-009-0432), with the main peaks located at 2θ ≈ 25.879° (002), 31.773° (211), 32.196° (112), and 32.902° (300). These reflections are consistent with a hexagonal hydroxyapatite structure belonging to the P6_3_/m space group. Although no additional crystalline phase was readily distinguishable by visual inspection of the diffraction pattern, Rietveld refinement resolved a minor whitlockite contribution, as summarized in Supplementary Table S1.

In the BHAp-TiO_2_ composite, three crystalline phases were identified: hydroxyapatite (PDF#00-009-0432), rutile TiO_2_ (PDF#00-021-1276), and whitlockite (PDF#00-009-0169). The rutile phase was identified by reflections at approximately 27.446° (110), 36.085° (101), 41.225° (111), 54.322° (211), 56.640° (220), and 69.008° (301), corresponding to a tetragonal structure with the P4_2_/mnm space group. The presence of these reflections indicates that TiO_2_ was present as an independent crystalline phase in the BHAp-TiO_2_ composite.

Additional diffraction signals assigned to whitlockite were detected at approximately 27.769° (214), 31.026° (0, 2, 10), and 34.371° (220). Since these reflections were not observed in the initial BHAp pattern, the appearance of whitlockite in BHAp-TiO_2_ suggests partial transformation of the calcium phosphate matrix during high-energy mechanochemical processing. This behavior is consistent with the structural disorder, lattice defects, and localized energy input generated during mechanical milling, which may promote phase rearrangement in calcium phosphate-based powders.

Rietveld refinement was performed to validate the phase composition of BHAp and BHAp-TiO_2_. The observed and calculated diffraction profiles, together with the difference curves and Bragg reflection positions, are shown in Figure 1C,D. The corresponding refinement parameters are summarized in Supplementary Table S1. For BHAp-TiO_2_, the refined phase fractions were 56.95 ± 0.83 wt% hydroxyapatite, 22.35 ± 0.61 wt% whitlockite, and 20.70 ± 0.38 wt% rutile TiO_2_. No anatase phase was detected within the XRD/Rietveld detection limit. The relatively high Bragg R-factor obtained for whitlockite suggests structural disorder or microstrain associated with the secondary calcium phosphate phase generated during mechanical milling.

The absence of relevant shifts in the hydroxyapatite reflections indicates that TiO_2_ was not incorporated into the hydroxyapatite lattice through Ti^4+^ substitution. Instead, the diffraction and refinement results support the formation of a multiphase BHAp-TiO_2_ ceramic composite composed of a hydroxyapatite-based calcium phosphate matrix associated with independent rutile TiO_2_ and whitlockite crystalline phases.

#### 3.1.2. Raman Spectroscopy

The Raman spectra of BHAp and BHAp-TiO_2_, recorded in the 100–1100 cm^−1^ range, are shown in Figure 1B. The BHAp spectrum exhibited the characteristic vibrational response of hydroxyapatite, dominated by the intense ν_1_(PO_4_^3−^) band near 960 cm^−1^. Additional bands located near 400, 600, and 1040 cm^−1^ were associated with phosphate bending and stretching modes of PO_4_^3−^ groups. For BHAp-TiO_2_, the Raman spectrum showed a marked decrease in the relative intensity of the phosphate-related bands, together with the appearance of additional TiO_2_-related vibrational bands. These spectral features support the presence of a TiO_2_-containing phase in the composite. The TiO_2_-related Raman response in BHAp-TiO_2_ was interpreted in conjunction with the XRD and Rietveld refinement results, which identified rutile as the crystalline TiO_2_ phase detected in the composite. The observed differences in Raman band shape and relative intensity may be associated with the effects of high-energy mechanochemical processing on the TiO_2_-containing phase, including crystallite-size effects, lattice strain, and structural disorder. Accordingly, Raman spectroscopy was considered complementary evidence of TiO_2_-related vibrational features, whereas the crystalline polymorphic assignment of the TiO_2_ phase was based primarily on XRD/Rietveld refinement. These results support the formation of a BHAp-TiO_2_ ceramic composite containing rutile TiO_2_ as an independent crystalline phase.

#### 3.1.3. Fourier-Transform Infrared Spectroscopy (FTIR)

The FTIR spectra of BHAp and BHAp-TiO_2_ are presented in Figure 1E. The BHAp spectrum showed the characteristic vibrational bands of a hydroxyapatite-based calcium phosphate material, including phosphate, hydroxyl, and carbonate-related contributions. The main phosphate absorption band located near 1020 cm^−1^ was assigned to the asymmetric stretching vibration of PO_4_^3−^ groups, while additional bands at approximately 1084 and 960 cm^−1^ were associated with phosphate stretching modes. Bands in the 560–630 cm^−1^ region were attributed to phosphate bending vibrations and hydroxyl-related contributions. The hydroxyl stretching band was observed near 3573 cm^−1^. Carbonate-related bands were identified at approximately 1412, 1453, and 873 cm^−1^, consistent with carbonated or non-stoichiometric apatite environments commonly observed in biogenic hydroxyapatite.

After mechanochemical processing with TiO_2_, the BHAp-TiO_2_ spectrum retained the characteristic phosphate bands of the calcium phosphate matrix, indicating preservation of the apatite-related vibrational framework. However, changes in band intensity and the appearance of additional shoulders in the phosphate region, particularly around 1125, 983, and 947 cm^−1^, suggest modifications in the local phosphate environment. These spectral changes are consistent with the multiphase nature of BHAp-TiO_2_ identified by XRD/Rietveld refinement, including the presence of hydroxyapatite and whitlockite-related calcium phosphate phases.

In the BHAp-TiO_2_ spectrum, a broad absorption contribution in the 660–880 cm^−1^ region was also observed and assigned to Ti–O and Ti–O–Ti vibrational modes associated with the TiO_2_-containing phase. This feature supports the presence of titanium oxide-related vibrational contributions in the composite, in agreement with the rutile TiO_2_ phase identified by XRD/Rietveld refinement. Overall, the FTIR results confirm the coexistence of phosphate-based vibrational modes from the calcium phosphate matrix and Ti–O-related contributions from the TiO_2_ phase, supporting the formation of a multiphase BHAp-TiO_2_ ceramic composite.

### 3.2. Surface Morphology and Elemental Composition

#### 3.2.1. Scanning Electron Microscopy (SEM)

**Figure 2:**
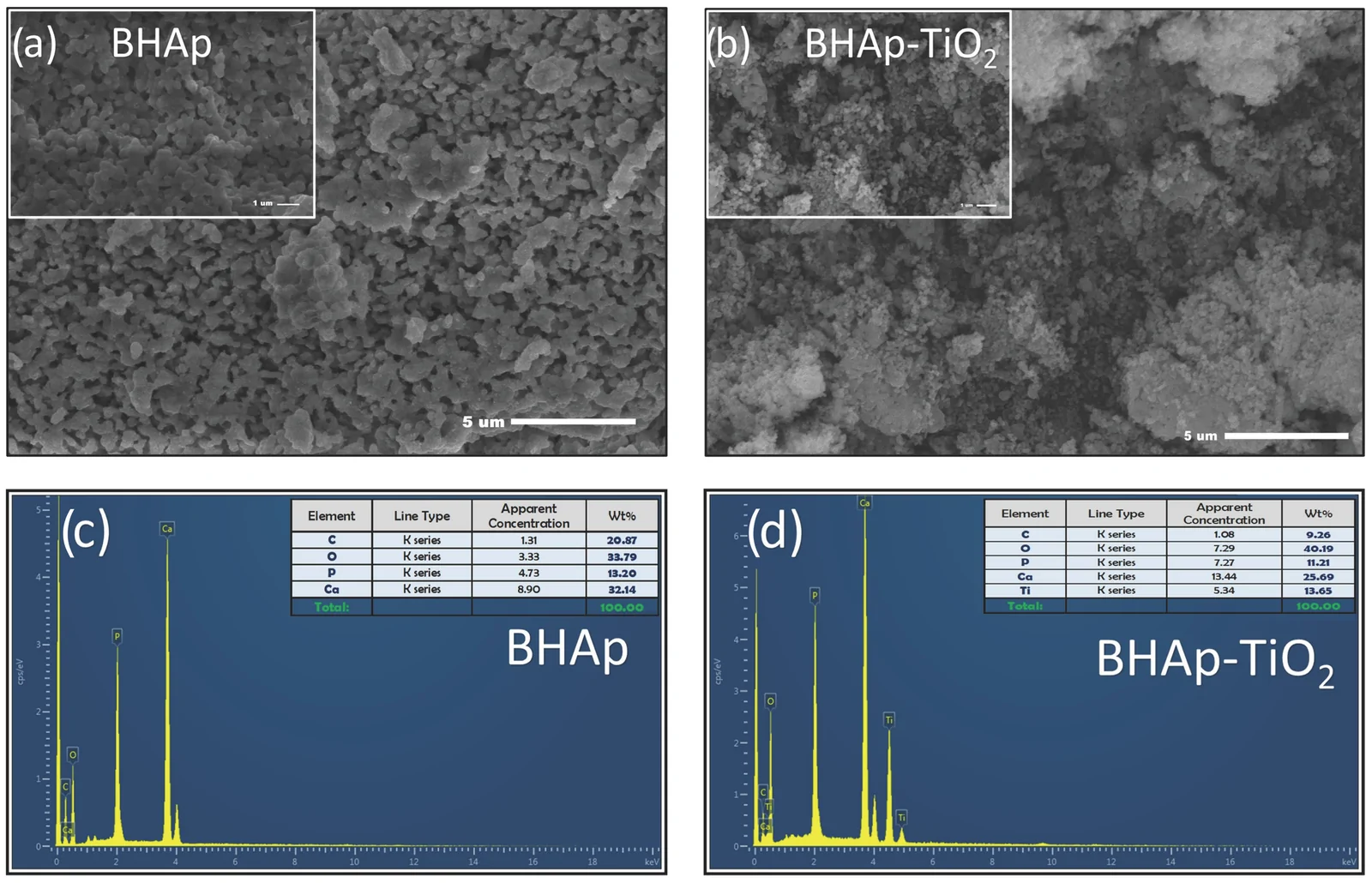
SEM morphology and EDS elemental analysis of BHAp and BHAp-TiO_2_ powders. (**a**,**b**) Representative SEM micrographs showing the surface morphology of BHAp and BHAp-TiO_2_, respectively. Insets show higher-magnification regions of the corresponding samples. The higher-magnification images were acquired independently from representative regions of the corresponding samples and do not necessarily represent direct digital enlargements of the highlighted areas (**c**,**d**) Representative EDS spectra of BHAp and BHAp-TiO_2_, respectively. EDS analysis confirmed the presence of Ca, P, and O in BHAp, whereas Ti was additionally detected in the BHAp-TiO_2_ sample.

Representative SEM micrographs of BHAp and BHAp-TiO_2_ are shown in Figure 2a,b. BHAp displayed an irregular granular morphology composed of heterogeneously sized particles assembled into micrometric agglomerates. At higher magnification, these agglomerates appeared to consist of smaller submicrometric particulate units with irregular contours, compact contact zones, and interparticle spaces distributed throughout the powder surface. The particles did not exhibit a defined geometric shape, but rather a fragmnted and heterogeneous morphology consistent with bone-derived calcium phosphate powders subjected to thermal processing and milling.

The BHAp surface showed a textured and uneven appearance, with closely packed particulate domains and localized discontinuities between adjacent granules. This morphology indicates a highly aggregated powder structure, in which smaller calcium phosphate particles are clustered into larger secondary agglomerates. Irregular particle boundaries and intergranular spaces were consistently observed across the analyzed regions.

Compared with BHAp, BHAp-TiO_2_ retained a granular and agglomerated architecture but exhibited a more compact and heterogeneous microstructure. The composite showed denser particle clusters, irregularly distributed fine particulate features, and local contrast variations across the agglomerate surface. These features indicate morphological modification after mechanochemical processing with TiO_2_, resulting in a composite powder with greater surface heterogeneity than pristine BHAp.

The morphological differences observed in BHAp-TiO_2_ are consistent with the effects of high-energy mechanochemical processing, including particle fracture, re-agglomeration, and structural rearrangement. These SEM observations describe qualitative changes in particle morphology and surface heterogeneity; quantitative roughness, porosity, or particle-size distribution measurements were not derived from these micrographs.

Overall, SEM analysis showed that both materials presented aggregated particulate morphologies, with BHAp-TiO_2_ displaying more compact agglomerates and a more heterogeneous surface appearance relative to BHAp. These morphological observations are consistent with the formation of a structurally distinct BHAp-TiO_2_ ceramic composite while remaining characteristic of biogenic hydroxyapatite-based powders.

#### 3.2.2. Energy Dispersive Spectroscopy (EDS)

The elemental composition of BHAp and BHAp-TiO_2_ powders was evaluated by SEM-EDS analysis (Figure 2c,d). The BHAp spectrum showed the characteristic signals of calcium (Ca), phosphorus (P), and oxygen (O), consistent with a calcium phosphate-based composition. In the BHAp-TiO_2_ sample, an additional titanium (Ti) signal was detected, confirming the elemental presence of Ti in the composite.

Semi-quantitative EDS analysis showed an increase in oxygen content from 33.79 wt% in BHAp to 40.19 wt% in BHAp-TiO_2_, accompanied by a relative decrease in Ca and P contents. This compositional variation is consistent with the contribution of the TiO_2_ phase to the overall elemental profile of the composite. The apparent Ca/P atomic ratio was approximately 1.88 for BHAp and 1.79 for BHAp-TiO_2_, both exceeding the theoretical Ca/P ratio of stoichiometric hydroxyapatite (1.67). These values indicate a calcium-rich, non-stoichiometric apatite composition in both materials.

Overall, SEM-EDS confirmed the expected calcium phosphate elemental profile of BHAp and the additional presence of Ti in BHAp-TiO_2_, supporting the formation of a TiO_2_-containing biogenic hydroxyapatite ceramic composite.

### 3.3. In Vitro Cytocompatibility Assessment

**Figure 3:**
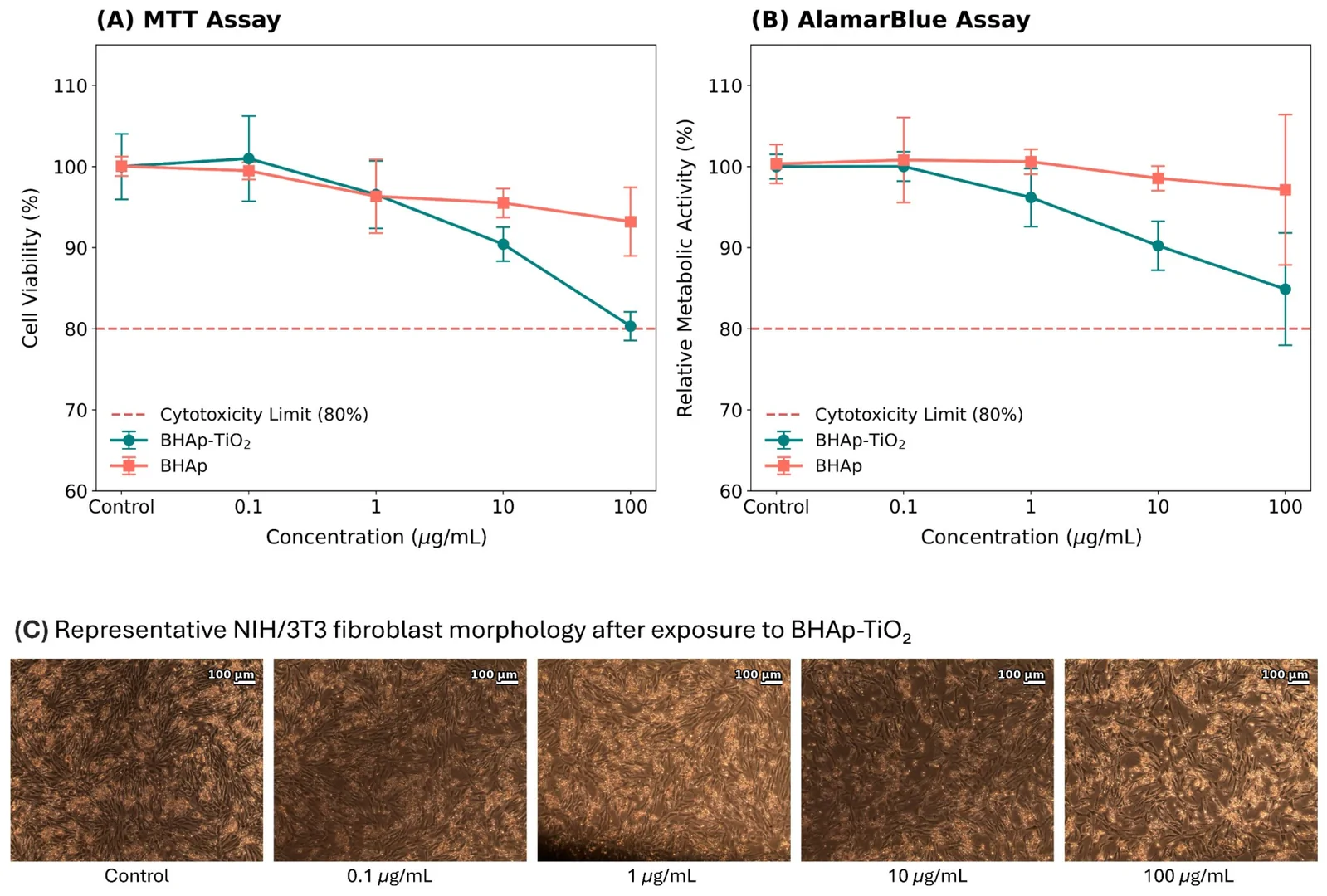
In vitro cytocompatibility of BHAp and BHAp-TiO_2_ in NIH/3T3 fibroblasts after 24 h of direct exposure. (**A**) Cell viability determined by MTT assay at 570 nm. (**B**) Relative metabolic activity determined by AlamarBlue^®^ assay using dual-wavelength absorbance correction at 570 and 600 nm. The dashed line indicates the 80% cytotoxicity limit used as an interpretative reference. Data are expressed as mean ± standard deviation from three independent experiments, with technical triplicates averaged within each independent experiment. (**C**) Representative optical microscopy images of NIH/3T3 fibroblast morphology after exposure to BHAp-TiO_2_. Images were acquired at 20× magnification.

The short-term cytocompatibility of BHAp and BHAp-TiO_2_ was evaluated in NIH/3T3 fibroblasts after 24 h of direct exposure using MTT and AlamarBlue^®^ assays (Figure 3A,B). The MTT assay showed that BHAp maintained high cell viability throughout the evaluated concentration range, with values of 99.48 ± 1.85%, 96.34 ± 7.89%, 95.51 ± 3.09%, and 93.21 ± 7.32% at 0.1, 1, 10, and 100 µg/mL, respectively. In comparison, BHAp-TiO_2_ showed a concentration-dependent reduction in MTT-derived cell viability, with values of 100.98 ± 9.09%, 96.54 ± 7.20%, 90.42 ± 3.63%, and 80.32 ± 3.06% at the same concentrations. Although the lowest viability was observed for BHAp-TiO_2_ at 100 µg/mL, the value remained close to the predefined 80% cytotoxicity limit. Significant differences between BHAp and BHAp-TiO_2_ were detected at 10 µg/mL (*p* = 0.018) and 100 µg/mL (*p* = 0.001). The AlamarBlue^®^ assay showed a similar concentration-dependent trend in relative metabolic activity. BHAp-treated fibroblasts maintained metabolic activity values close to untreated control levels, with 100.80 ± 9.08%, 100.59 ± 2.65%, 98.56 ± 2.62%, and 97.14 ± 16.06% at 0.1, 1, 10, and 100 µg/mL, respectively. In contrast, BHAp-TiO_2_ produced a gradual decrease in metabolic activity, with values of 100.02 ± 3.11%, 96.18 ± 6.19%, 90.24 ± 5.23%, and 84.88 ± 11.99% across the same concentration range. These results indicate that, within the evaluated 24-h exposure period, BHAp-TiO_2_ reduced fibroblast metabolic activity in a concentration-dependent manner while remaining above the 80% cytotoxicity limit. Representative optical microscopy images further supported the quantitative findings (Figure 3C). NIH/3T3 fibroblasts exposed to BHAp-TiO_2_ retained an adherent fibroblast-like morphology across the tested concentrations. Cells showed elongated and spindle-shaped features characteristic of fibroblastic cultures, without evident extensive detachment or severe morphological disruption after 24 h of exposure. These observations are consistent with the preservation of short-term cytocompatibility within the evaluated concentration range.

### 3.4. Antimicrobial Activity Against Planktonic Bacteria

**Figure 4:**
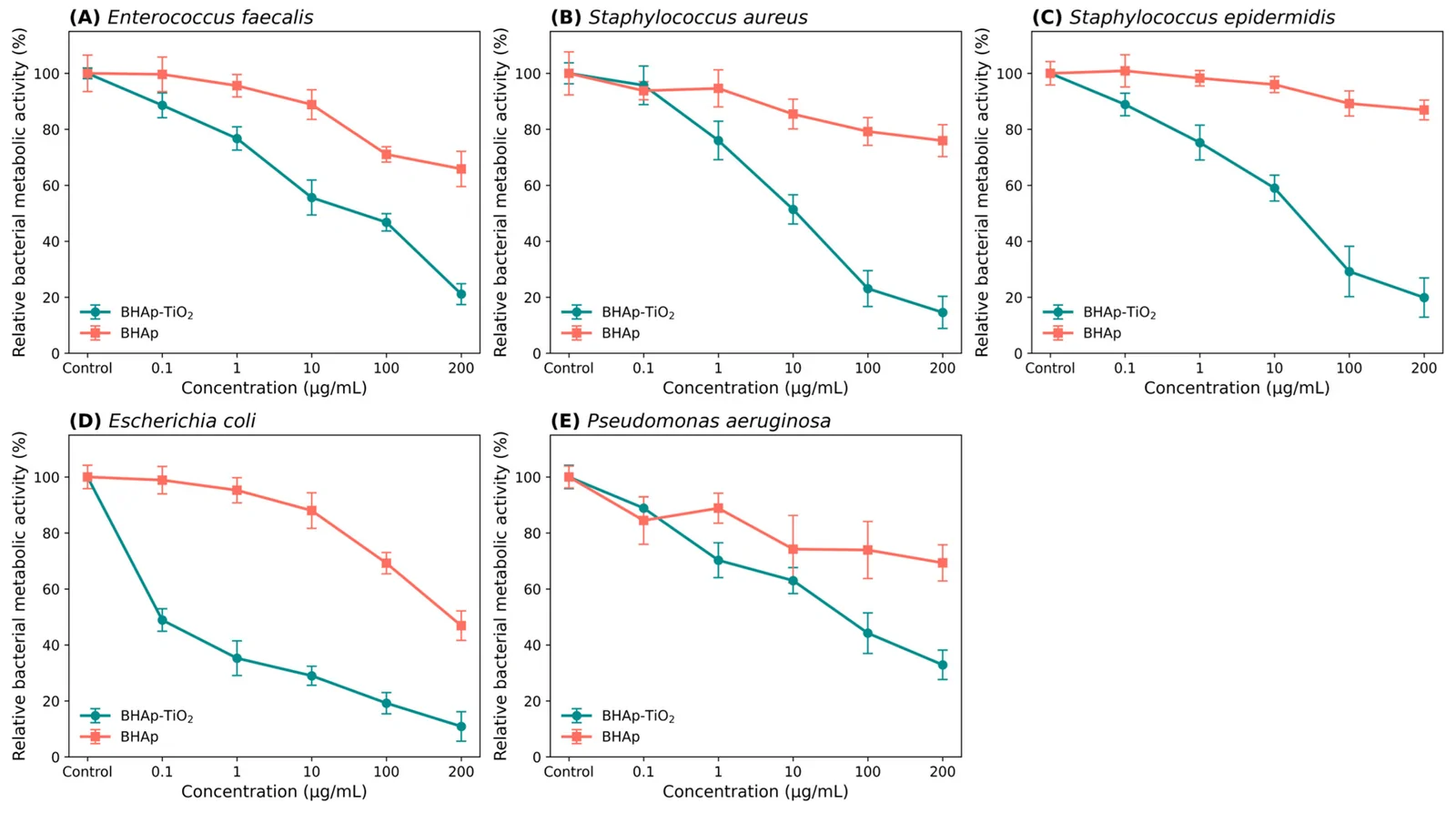
Antimicrobial effect of BHAp and BHAp-TiO_2_ against planktonic bacteria evaluated by the AlamarBlue^®^ assay after 24 h of exposure. Relative bacterial metabolic activity was determined for (**A**) *Enterococcus faecalis*, (**B**) *Staphylococcus aureus*, (**C**) *Staphylococcus epidermidis*, (**D**) *Escherichia coli*, and (**E**) *Pseudomonas aeruginosa*. Untreated bacterial cultures were defined as 100% metabolic activity. Data are expressed as mean ± standard deviation from three independent experiments, with technical triplicates averaged within each independent experiment.

The antimicrobial effect of BHAp and BHAp-TiO_2_ against planktonic bacteria was evaluated using the AlamarBlue^®^ metabolic assay after 24 h of exposure. As shown in Figure 4A–E, untreated bacterial cultures were defined as 100% relative bacterial metabolic activity. BHAp produced a limited to moderate reduction in metabolic activity depending on the bacterial strain and concentration, whereas BHAp-TiO_2_ induced a more pronounced concentration-dependent decrease in metabolic activity across all evaluated microorganisms.

Among Gram-positive strains, BHAp-TiO_2_ reduced the metabolic activity of *Enterococcus faecalis* from 88.60 ± 4.42% at 0.1 µg/mL to 21.14 ± 3.73% at 200 µg/mL. For *Staphylococcus aureus*, metabolic activity decreased from 95.74 ± 6.90% at 0.1 µg/mL to 14.60 ± 5.71% at 200 µg/mL. A similar response was observed for *Staphylococcus epidermidis*, in which BHAp-TiO_2_ reduced metabolic activity from 88.89 ± 4.02% at 0.1 µg/mL to 19.90 ± 7.00% at 200 µg/mL. In contrast, BHAp showed a substantially weaker effect, with residual metabolic activity values of 65.86 ± 6.30%, 75.96 ± 5.69%, and 86.90 ± 3.53% at 200 µg/mL for *E. faecalis*, *S. aureus*, and *S. epidermidis*, respectively (Figure 4).

The Gram-negative strains also exhibited a concentration-dependent response to BHAp-TiO_2_. *Escherichia coli* was the most sensitive strain, showing a marked reduction in metabolic activity even at 0.1 µg/mL, with values decreasing from 48.89 ± 4.02% at 0.1 µg/mL to 10.90 ± 5.26% at 200 µg/mL. *Pseudomonas aeruginosa* showed a comparatively lower response, although BHAp-TiO_2_ still reduced metabolic activity from 88.89 ± 4.02% at 0.1 µg/mL to 32.90 ± 5.26% at 200 µg/mL. At the same highest concentration, BHAp-treated cultures retained higher metabolic activity, with values of 46.90 ± 5.26% for *E. coli* and 69.32 ± 6.46% for *P. aeruginosa* (Figure 4).

At 200 µg/mL, BHAp-TiO_2_ produced the strongest AlamarBlue^®^-derived antimicrobial response, reducing bacterial metabolic activity to 21.14 ± 3.73% in *E. faecalis*, 14.60 ± 5.71% in *S. aureus*, 19.90 ± 7.00% in *S. epidermidis*, 10.90 ± 5.26% in *E. coli*, and 32.90 ± 5.26% in *P. aeruginosa*. These values correspond to metabolic inhibition levels of 78.86%, 85.40%, 80.10%, 89.10%, and 67.10%, respectively.

Statistical analysis showed significant differences between BHAp and BHAp-TiO_2_ at all evaluated concentrations from 1 to 200 µg/mL for *E. faecalis*, *S. aureus*, *S. epidermidis*, and *P. aeruginosa* (*p* < 0.05). For *E. coli*, significant differences between both materials were detected from 0.1 to 200 µg/mL (*p* < 0.05). Overall, these results indicate that the presence of TiO_2_ in BHAp-TiO_2_ markedly enhanced the antimicrobial effect of the biogenic hydroxyapatite-based material, with a strain-dependent response and the highest activity observed against *E. coli*, *S. aureus*, and *S. epidermidis*.

### 3.5. Antibiofilm Activity Against Pseudomonas aeruginosa

**Figure 5:**
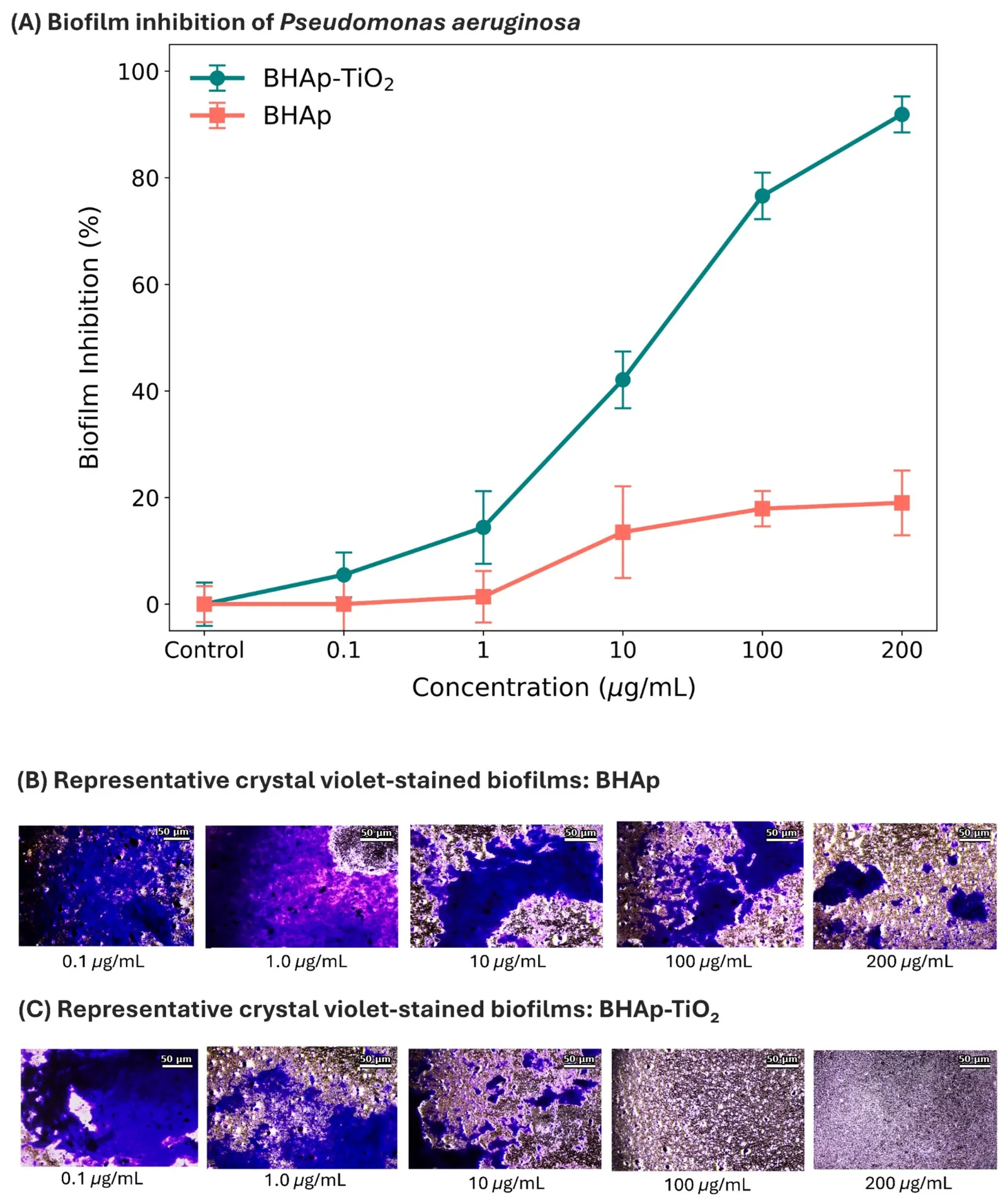
Antibiofilm effect of BHAp and BHAp-TiO_2_ against *Pseudomonas aeruginosa* evaluated by crystal violet staining after 24 h of static biofilm formation. (**A**) Biofilm inhibition calculated from blank-corrected absorbance values at 570 nm. Untreated biofilms were defined as 0% inhibition. Data are expressed as mean ± standard deviation from three independent experiments, with technical triplicates averaged within each independent experiment. (**B**,**C**) Representative optical microscopy images of crystal violet-stained biofilms exposed to BHAp and BHAp-TiO_2_, respectively. All subfigures correspond to independent microscopic fields. Images were acquired at 20× magnification.

The antibiofilm activity of BHAp and BHAp-TiO_2_ against *Pseudomonas aeruginosa* was evaluated using the crystal violet staining assay after 24 h of static biofilm formation. Biofilm inhibition was calculated from blank-corrected absorbance values and expressed relative to untreated biofilm controls, which were defined as 0% inhibition. As shown in Figure 5A, BHAp-TiO_2_ produced a marked concentration-dependent antibiofilm effect, whereas BHAp showed only limited inhibition across the evaluated concentration range.

At low concentrations, BHAp-TiO_2_ produced modest biofilm inhibition, with values of 5.5 ± 4.2% at 0.1 µg/mL and 14.4 ± 6.8% at 1 µg/mL. A stronger inhibitory response was observed from 10 µg/mL onward, reaching 42.1 ± 5.3% inhibition at 10 µg/mL, 76.6 ± 4.4% at 100 µg/mL, and 91.9 ± 3.4% at 200 µg/mL. In contrast, BHAp exhibited a substantially lower antibiofilm effect, with inhibition values of 0.0 ± 5.4%, 1.4 ± 4.8%, 13.5 ± 8.6%, 17.9 ± 3.3%, and 19.0 ± 6.1% at 0.1, 1, 10, 100, and 200 µg/mL, respectively.

These results indicate that the presence of TiO_2_ in BHAp-TiO_2_ substantially enhanced the antibiofilm performance of the biogenic hydroxyapatite-based material. At the highest tested concentration, BHAp-TiO_2_ reduced the relative biofilm biomass to 8.1%, corresponding to 91.9% inhibition, whereas BHAp retained 81.0% relative biofilm biomass, corresponding to only 19.0% inhibition (Figure 5A).

The qualitative microscopy images of crystal violet-stained biofilms were consistent with the quantitative results (Figure 5B,C). Biofilms exposed to BHAp retained extensive crystal violet staining across most concentrations, indicating persistent surface-associated biomass. In contrast, BHAp-TiO_2_-treated biofilms showed a progressive reduction in stained biomass with increasing concentration, particularly at 100 and 200 µg/mL, where lower staining intensity and reduced surface coverage were evident. These observations support the concentration-dependent antibiofilm effect of BHAp-TiO_2_ against *P. aeruginosa* under the evaluated static culture conditions.

## 4. Discussion

The development of infection-resistant biomaterials remains a major challenge in regenerative medicine, particularly for bone-repair and implant-associated applications, where bacterial adhesion, planktonic proliferation, and biofilm formation can compromise tissue integration and clinical performance [1,2,21]. Hydroxyapatite-based materials are attractive for bone-related applications because of their chemical affinity with the mineral phase of bone and their osteoconductive potential; however, hydroxyapatite alone does not provide broad-spectrum antimicrobial protection [7,22]. Therefore, composite calcium phosphate systems incorporating antimicrobial oxide phases, such as TiO_2_, have emerged as promising strategies to improve bacterial control while preserving biological compatibility [12,14,15].

### 4.1. Structural and Phase-Related Implications of the BHAp-TiO_2_ Ceramic Composite

The structural characterization redefined the interpretation of BHAp-TiO_2_. XRD and Rietveld refinement showed that TiO_2_ was present as an independent rutile crystalline phase associated with a hydroxyapatite-based calcium phosphate matrix, rather than as Ti^4+^ incorporated into the hydroxyapatite lattice. This distinction is relevant because ion-substituted hydroxyapatites and ceramic composites may differ substantially in phase stability, surface chemistry, dissolution behavior, interfacial reactivity, and bacteria–material interactions [10]. Accordingly, the biological responses observed in this study should be interpreted in the context of a multiphase ceramic composite rather than a Ti-doped hydroxyapatite system.

The formation of whitlockite in BHAp-TiO_2_ further indicates that high-energy mechanochemical processing modified the calcium phosphate matrix. Planetary milling can induce particle fracture, re-agglomeration, lattice defects, microstrain, and local solid-state rearrangements, which may promote secondary calcium phosphate phase formation [23]. This structural interpretation is consistent with the SEM observations, where BHAp-TiO_2_ exhibited denser agglomerates and greater morphological heterogeneity than pristine BHAp. However, because roughness, porosity, particle-size distribution, and depth-dependent Ti distribution were not quantified, these morphological observations should be interpreted qualitatively.

The Raman and FTIR results support the multiphase interpretation when considered together with XRD/Rietveld refinement. Raman spectroscopy revealed TiO_2_-related vibrational features in BHAp-TiO_2_, whereas FTIR showed the coexistence of phosphate-based bands and Ti–O/Ti–O–Ti contributions. Since Raman spectroscopy is sensitive to local bonding environments, crystallite size, lattice strain, and structural disorder, the TiO_2_ polymorphic assignment was based primarily on XRD/Rietveld refinement, while Raman and FTIR were interpreted as complementary vibrational evidence. Overall, the characterization supports the formation of a BHAp-TiO_2_ ceramic composite composed of a hydroxyapatite-rich matrix associated with rutile TiO_2_ and whitlockite phases.

### 4.2. Short-Term Cytocompatibility and Cellular Response

BHAp and BHAp-TiO_2_ were evaluated in NIH/3T3 fibroblasts as a preliminary model of mammalian cell response to direct material exposure. The combined use of MTT and AlamarBlue^®^ assays provided complementary information on cell viability and metabolic activity, respectively, strengthening the interpretation of short-term cytocompatibility [24]. BHAp maintained cellular responses close to untreated control levels, consistent with the widely reported compatibility of calcium phosphate-based biomaterials [7,22].

BHAp-TiO_2_ produced a concentration-dependent reduction in fibroblast response, particularly at the highest concentrations evaluated; however, values remained close to or above the 80% cytotoxicity limit used as an interpretative reference. This moderate decrease may reflect the combined effect of the TiO_2_-containing phase, altered particle morphology, local cell–material contact, and sedimentation effects inherent to direct-contact assays with particulate ceramics [25]. Therefore, the observed response should not be interpreted as overt cytotoxicity, but rather as a concentration-dependent modulation of cellular metabolic activity under short-term exposure conditions.

The presence of rutile TiO_2_ may be relevant to this response. Rutile is generally considered more thermodynamically stable and less photocatalytically reactive than anatase, which may contribute to a more moderate biological reactivity profile [12]. Nevertheless, oxidative stress, mitochondrial dysfunction, membrane damage, and inflammatory mediators were not directly measured; therefore, the role of rutile in preserving fibroblast cytocompatibility remains mechanistic and requires further validation. Likewise, optical microscopy supported the absence of severe morphological disruption, but it does not replace quantitative cytoskeletal, apoptosis/necrosis, proliferation, or inflammatory assays. Thus, the present findings support short-term fibroblast cytocompatibility within the evaluated 0.1–100 µg/mL range, but not comprehensive long-term biocompatibility.

### 4.3. Antimicrobial Performance Against Planktonic Bacteria

BHAp-TiO_2_ showed a stronger inhibitory effect on bacterial metabolic activity than pristine BHAp across all evaluated planktonic strains. This response was concentration-dependent and strain-dependent, with the most pronounced metabolic reductions observed at 200 µg/mL. The enhanced antimicrobial response should be interpreted in relation to the composite architecture of BHAp-TiO_2_, where rutile TiO_2_ exists as an independent crystalline phase associated with the calcium phosphate matrix. Therefore, the activity is more appropriately attributed to composite-mediated bacteria–material interactions than to Ti^4+^ substitution within the apatite lattice.

The antimicrobial behavior of BHAp-TiO_2_ is consistent with reports describing improved bacterial inhibition in hydroxyapatite/TiO_2_-based systems [14,15]. TiO_2_-containing materials may affect bacteria through oxidative stress, membrane perturbation, disruption of respiratory metabolism, surface charge interactions, and impaired adhesion [12,26]. However, these mechanisms were not directly quantified in the present work. Because AlamarBlue^®^ measures resazurin reduction as an indicator of metabolic activity, the observed decrease reflects metabolic suppression rather than direct evidence of bactericidal activity. Consequently, log_10_ CFU/mL reductions were not derived from these data.

Rare-earth-containing systems represent an alternative strategy for modifying hydroxyapatite-rich materials and provide a relevant comparison with the present BHAp–TiO_2_ composite [9,10]. The physicochemical and biological responses of these systems depend on the identity and chemical form of the incorporated element, its concentration, ion-release behavior, and interaction with the mineral substrate [9,10]. In this context, Kopp et al. demonstrated that cerium(III) and samarium(III) nitrates precipitated on human enamel independently of the presence of a salivary pellicle. EDX analysis confirmed surface-associated Ce and Sm and showed that several treatments significantly decreased the enamel Ca/P ratio compared with the untreated controls [10]. These findings demonstrate the capacity of rare-earth compounds to interact with hydroxyapatite-rich mineralized surfaces. Nevertheless, these soluble lanthanide formulations differ fundamentally from the material investigated in the present study, in which rutile TiO_2_ remains an independent crystalline phase associated with a biogenic calcium phosphate matrix. Consequently, the antimicrobial activity and cytocompatibility of these material classes cannot be directly extrapolated and must be interpreted according to their specific phase composition, ion-release behavior, and concentration-dependent biological response.

The strain-dependent response was not governed solely by Gram classification. Although *Staphylococcus aureus* and *Staphylococcus epidermidis* were highly susceptible, *Escherichia coli* exhibited the strongest reduction in metabolic activity, whereas *Pseudomonas aeruginosa* retained the highest residual activity among the BHAp-TiO_2_-treated groups. This behavior reflects differences in envelope structure, surface charge, metabolic state, stress-response capacity, and the probability of direct contact with particulate material. The lower susceptibility of *P. aeruginosa* is consistent with its intrinsic tolerance mechanisms, including low outer-membrane permeability, efflux systems, oxidative stress defenses, metabolic adaptability, and strong biofilm-forming capacity [27].

Although comparison with BHAp demonstrates the contribution of the TiO_2_-containing phase to antimicrobial performance, the absence of a TiO_2_-only control limits mechanistic deconvolution. Therefore, the present results support enhanced antimicrobial activity of BHAp-TiO_2_ relative to BHAp, but do not determine whether this effect is dominated by rutile TiO_2_ itself or by calcium phosphate/TiO_2_ interfacial interactions. Future studies should include TiO_2_-only controls, viable colony counts, membrane integrity assays, and reactive oxygen species measurements to distinguish bacteriostatic, bactericidal, and metabolism-suppressive effects.

### 4.4. Antibiofilm Activity Against Pseudomonas aeruginosa

The antibiofilm assay demonstrated that BHAp-TiO_2_ strongly inhibited *P. aeruginosa* biofilm biomass in a concentration-dependent manner. After correction of the biofilm inhibition calculation, untreated biofilms were defined as 0% inhibition, and BHAp-TiO_2_ produced its highest inhibitory response at 200 µg/mL. In contrast, BHAp showed only limited antibiofilm activity, indicating that the TiO_2_-containing phase substantially improved the ability of the biogenic hydroxyapatite matrix to interfere with biofilm biomass accumulation.

This finding is biologically relevant because biofilm-associated bacteria differ markedly from planktonic cells in metabolic activity, matrix production, spatial organization, antimicrobial tolerance, and immune evasion [28,29]. The use of *P. aeruginosa* as a focused antibiofilm model is justified by its robust biofilm-forming capacity and relevance in chronic wounds and device-associated infections [1,30]. Nevertheless, this should be interpreted as targeted antibiofilm screening rather than broad-spectrum biofilm validation.

The crystal violet assay quantifies total attached biomass and does not distinguish viable cells from extracellular polymeric matrix or non-viable biomass [31]. Thus, the reduced staining observed after BHAp-TiO_2_ exposure should be interpreted as decreased surface-associated biofilm biomass, not as direct evidence of biofilm killing. The qualitative microscopy images were consistent with this interpretation, showing reduced staining intensity and lower surface coverage at higher BHAp-TiO_2_ concentrations. However, biofilm thickness, three-dimensional structure, EPS composition, and spatial viability were not resolved.

The stronger antibiofilm effect of BHAp-TiO_2_ compared with BHAp may be related to the heterogeneous ceramic architecture of the composite. The coexistence of calcium phosphate and rutile TiO_2_ domains may alter bacterial adhesion, interfacial contact, and matrix accumulation during early biofilm development. However, the current data do not distinguish between inhibition of initial adhesion, disruption of microcolony development, reduced EPS accumulation, or detachment of weakly adhered biomass. Future studies using viable biofilm counts, live/dead confocal microscopy, EPS-specific staining, and clinically derived mono- or multispecies biofilms will be required to define the antibiofilm mechanism.

### 4.5. Limitations and Future Perspectives

Several limitations should be considered. First, the biological assays were performed using particulate powders under direct-contact or suspension-based conditions. Therefore, the results cannot be directly extrapolated to immobilized coatings, scaffolds, or implant-associated surfaces, where particle distribution, surface exposure, mechanical stability, and interfacial contact with cells or bacteria may differ substantially. Future studies should evaluate BHAp-TiO_2_ in surface-stabilized configurations that better reproduce clinically relevant bone-related biomaterial interfaces. Second, surface roughness, porosity, and particle-size distribution were not quantitatively measured. Although SEM analysis revealed morphological heterogeneity and compact agglomeration in BHAp-TiO_2_, quantitative topographical and textural analyses will be required to correlate surface architecture with bacterial adhesion, biofilm formation, and cellular response. Third, cytocompatibility was evaluated only after 24 h and within the 0.1–100 µg/mL concentration range, whereas antimicrobial and antibiofilm assays included 200 µg/mL. Thus, cytocompatibility at the highest antimicrobial concentration remains to be determined. Longer exposure periods, including 48 h, 72 h, and extended culture models, should be incorporated to more comprehensively define the biological safety window of the composite. Fourth, direct exposure to particulate powders may introduce sedimentation, localized cell–material contact, and optical interference in colorimetric or metabolic assays. Complementary extract-based assays, material-only optical controls, and additional cellular models, including osteoblasts, mesenchymal stromal cells, endothelial cells, and macrophage-related inflammatory models, should be included in future work.

An additional limitation concerns the experimental sample size and the resulting statistical power. No formal a priori sample-size calculation was performed, and only three independent experiments were available per condition. Consequently, biological results should be considered exploratory and require confirmation in prospectively powered studies incorporating a larger number of independent experiments.

Finally, the antimicrobial and antibiofilm assays relied on metabolic and biomass-based readouts rather than viable bacterial counts. Therefore, bactericidal activity and log_10_ CFU/mL reductions remain to be established. The absence of a TiO_2_-only control also limits the ability to determine whether the enhanced antimicrobial and antibiofilm responses arise primarily from rutile TiO_2_ itself or from composite-specific interactions at the calcium phosphate/TiO_2_ interface. Future studies should include TiO_2_-only controls, viable colony counts, live/dead biofilm imaging, EPS quantification, reactive oxygen species measurements, and clinically derived mono- or multispecies biofilm models.

## 5. Conclusions

This study demonstrates that high-energy mechanical milling enabled the formation of a multiphase BHAp-TiO_2_ ceramic composite composed of a biogenic hydroxyapatite-based calcium phosphate matrix associated with independent rutile TiO_2_ and whitlockite crystalline phases. The structural and spectroscopic analyses support the interpretation of BHAp-TiO_2_ as a ceramic composite rather than a Ti-doped hydroxyapatite system, since no evidence of Ti^4+^ substitution into the hydroxyapatite lattice was observed. Biologically, BHAp-TiO_2_ exhibited enhanced antimicrobial activity against clinically relevant planktonic bacteria and a marked concentration-dependent antibiofilm effect against *Pseudomonas aeruginosa* compared with pristine BHAp. At the same time, the composite preserved short-term fibroblast cytocompatibility within the evaluated concentration range, indicating a favorable balance between bacterial inhibition and mammalian cell response under the tested conditions. Overall, these findings position BHAp-TiO_2_ as a promising TiO_2_-containing biogenic calcium phosphate composite for the development of infection-resistant bone-related biomaterials. Further studies incorporating long-term cytocompatibility assays, osteogenic and inflammatory cell models, viable bacterial counts, TiO_2_-only controls, and surface-stabilized configurations will be necessary to clarify its mechanism of action, biological safety window, and translational potential.

## Data Availability

The original contributions presented in this study are included in the article. Further inquiries can be directed to the corresponding author.

## Supplementary Materials

The following supporting information can be downloaded at https://www.mdpi.com/article/10.3390/jfb17090426/s1, Table S1: Phase-specific Rietveld refinement parameters and weight fractions obtained for BHAp and BHAp-TiO_2_ powders.
