# Supplementary material for: Mechanochemical Synthesis of a TiO2-Containing Biogenic Hydroxyapatite Ceramic Composite: Balancing Antibiofilm Efficacy and Fibroblast Cytocompatibility
Source: J Funct Biomater. 2026 Aug 24;17(9):426. doi: 10.3390/jfb17090426 (PMC13608521; doi:10.3390/jfb17090426)
Supplement: Supplementary file 1 [file jfb-17-00426-s001.zip › jfb-4385258-supplementary.pdf]

Supplementary material

Table S1. Phase-specific Rietveld refinement parameters and weight fractions obtained for BHAp and BHAp-TiO_2 powders.

Sample	Refined phase	Space group	Unit-cell volume (Å³)	Bragg R-factor	Rf-factor	ATZ	Brindley factor	Weight fraction (wt%)
BHAp	Whitlockite	R3c	3457.233 ± 0.000	40.9	20.7	6634.070	1.0000	6.07 ± 0.32
BHAp	Hydroxyapatite (HAp-IA)	$P6_3/m$	530.009 ± 0.004	14.8	8.39	1004.632	1.0000	93.93 ± 0.82
BHAp-TiO_2	Whitlockite	R3c	3457.233 ± 0.153	24.3	14.9	6634.070	1.0000	22.35 ± 0.61
BHAp-TiO_2	Rutile TiO_2	$P4_2/mnm$	62.581 ± 0.002	7.81	4.58	159.732	1.0000	20.70 ± 0.38
BHAp-TiO_2	Hydroxyapatite (HAp-IA)	$P6_3/m$	530.021 ± 0.008	13.0	8.31	1004.632	1.0000	56.95 ± 0.83

Note. Phase fractions are reported as weight percentages according to the FullProf Rietveld refinement output. The values in parentheses from the refinement output were expressed as standard uncertainties. BHAp-TiO_2 showed a multiphase composition consisting of hydroxyapatite, whitlockite, and rutile TiO_2, with no anatase phase detected within the XRD/Rietveld detection limit.
